# Fusion of Transformer and RBF for Anomalous Traffic Detection in Sensor Networks

**DOI:** 10.3390/s26020515

**Published:** 2026-01-13

**Authors:** Aibing Dai, Jianwei Guo, Yuanyuan Hou, Yiou Wang

**Affiliations:** Beijing Academy of Science and Technology, Beijing 100089, China

**Keywords:** sensor network, abnormal traffic detection, fusion of radial basis functions

## Abstract

With the widespread adoption of the Internet of Things (IoT) and smart devices, the volume of data generated in sensor networks has increased dramatically, with diverse and structurally complex types that pose growing security risks. Anomaly detection in sensor networks has become a key technology for ensuring system stability and secure operation. This paper proposes a sensor anomaly detection model, termed RESTADM, which integrates a Transformer and a Radial Basis Function (RBF) neural network. The model first employs the Transformer to effectively capture the temporal dependencies in sensor data and then uses the RBF neural network to accurately identify anomalies. Experimental results on two public benchmark datasets, SMD and PSM, demonstrate the state-of-the-art performance of RESTADM. Our model achieves impressive F1-scores of 98.56% on SMD and 97.70% on PSM. This represents a statistically significant improvement compared to a range of baseline algorithms, including traditional models like CNN and LSTM, as well as the standard Transformer model. This validates the effectiveness of our proposed Transformer-RBF fusion, confirming the model’s high accuracy and robustness and offering an efficient security solution for intelligent sensing systems.

## 1. Introduction

With the rapid development of China’s Internet of Things (IoT) and intelligent sensing technologies, sensor networks have been widely deployed across critical domains such as industrial control, environmental monitoring, healthcare, and smart cities [1,2,3]. However, as the scale of sensor-device connectivity expands, the security threats to these systems have continued to intensify. According to the 49th issue of the Cybersecurity Information and Dynamics Weekly Report published by the National Internet Emergency Center (CNCERT) in 2024 [4], the number of backdoor-infected devices and systems in China increased by 47.8% year-on-year, while the propagation frequency of malicious programs in edge devices and embedded systems rose by 29.2%. The security landscape of sensor networks has become increasingly critical. Against this backdrop, sensor data anomaly detection, as a key technology for ensuring system security and stable operation, holds substantial research value and practical significance [5].

Sensor network data traffic consists of a collection of real-time data signals, acquired by numerous sensor nodes and transmitted through communication links. This data encompasses a diverse array of protocols and formats, reflecting communication patterns between nodes, variations in environmental conditions, and operational behaviors of devices [6]. In-depth analysis of sensor data not only enables the identification of potential faults or security vulnerabilities but also provides technical support for system optimization and anomaly detection mechanisms (where ‘anomaly’ is translated as ‘early warning’; see note below). Anomaly detection is a technique used to identify behaviors that significantly deviate from normal patterns. Its fundamental principle involves developing statistical or machine learning models using normal data, followed by the identification of behaviors that deviate from these models. In sensor networks, anomalies may arise from various factors such as equipment malfunctions, communication interference, or malicious attacks.

Early methods for network traffic anomaly detection primarily relied on manually engineered features and statistical analysis. Common statistical approaches assume that data follows a specific distribution and detect anomalies by quantifying deviations from this distribution. For instance, the Local Outlier Factor (LOF) algorithm detects anomalies by evaluating changes in data point density, while clustering-based algorithms classify data according to distance metrics. These methods perform well when dealing with low-dimensional data but often face substantial limitations when applied to high-dimensional and heterogeneous network traffic data. Additionally, since statistical methods fail to capture temporal information in traffic patterns, they are inadequate in dynamic network environments that demand stringent real-time responsiveness [7].

However, sensor data exhibits characteristics such as high dimensionality, unstructured nature, multi-source heterogeneity, and rapid, dynamic fluctuations. Traditional anomaly detection methods often struggle to manage complex data patterns under these conditions [8]. Early research predominantly relied on manually engineered features, in combination with statistical methods, including the Local Outlier Factor (LOF) and clustering analysis. While these approaches demonstrated satisfactory performance in low-dimensional settings, they struggled to effectively capture the nonlinear characteristics and temporal dependencies intrinsic to complex sensor data [9]. With the enhancement of computing power in sensing terminals and the accumulation of large-scale sensor data, machine learning methods have been extensively applied in the field of anomaly detection. Models such as Support Vector Machine (SVM), K-Nearest Neighbors (KNN), and Decision Trees have achieved moderate detection performance on small-scale datasets [10,11]. However, these approaches heavily rely on manual feature extraction, display limited generalization capabilities, and fail to adapt to dynamic and variable sensing environments [12]. Furthermore, when dealing with large-scale heterogeneous sensor data, these algorithms encounter significant bottlenecks in terms of training efficiency and computational resource requirements.

In addition to these models, RBF neural networks have also been explored for security applications due to their strong nonlinear mapping capabilities. Early research demonstrated the effectiveness of hybrid approaches; for instance, Tong et al. [13] used a hybrid RBF/Elman neural network for an intrusion detection system secure model. Further expanding on this, Zhang et al. [14] proposed a network security situation prediction model based on BP and RBF neural networks. More recently, researchers have continued to leverage RBF’s strengths in modern security frameworks; Lopez-Martin et al. [15] developed an intrusion detection system using an extended RBF network with offline reinforcement learning, enhancing its adaptability. These works highlight the ongoing relevance of RBF networks and provide a basis for our proposed fusion model. Previous studies have attempted to combine RBF with deep learning models for anomaly detection. However, these approaches typically appended an RBF layer at the end of the architecture, without exploring its synergy with Transformer-based temporal modeling. In contrast, RESTADM strategically places the RBF layer after the second encoder, which allows it to enhance local feature discrimination while preserving the Transformer’s ability to capture long-term dependencies.

In recent years, deep learning methods have gradually emerged as a prominent research focus in sensor anomaly detection, owing to their robust ability for automatic feature extraction. Convolutional Neural Networks (CNNs) excel at capturing spatial feature patterns, making them suitable for anomaly identification in static structured data; Recurrent Neural Networks (RNNs) and their Long Short-Term Memory (LSTM) architectures, on the other hand, excel at processing sequential data and modeling dynamic changes in sensor data [16]. The integration of these methods provides a theoretical foundation for precise detection in complex, variable sensor environments [17].

On this basis, the Transformer model, with its structural design based on self-attention mechanisms, has achieved groundbreaking results in natural language processing and time series modeling. Compared to traditional Recurrent Neural Networks, the Transformer offers parallel computing capabilities and effectively captures long-range dependencies, thereby demonstrating superior performance when processing continuous sensor data streams [18]. Its multi-head attention mechanism can simultaneously model local and global features, thus providing new avenues for anomaly detection in complex data environments.

Recently, Yuan et al. [19] proposed a hybrid and spatiotemporal detection framework (SCIS) for cyberattack network traffic in cloud data centers. Their model integrates CNNs, Informer, and Softmax classifiers to effectively capture spatiotemporal dependencies in network traffic flows, achieving significant improvements in detecting cyberattacks such as Denial-of-Service (DoS) and Distributed Denial-of-Service (DDoS). By leveraging CNNs to extract spatial features across network topologies and Informer to model temporal patterns in traffic sequences, their work provides valuable insights into hybrid modeling strategies for network-layer intrusion detection.

While both frameworks apply hybrid deep learning to anomaly detection, their primary application domains and data modalities differ significantly. Yuan et al. [19] specializes in identifying cyberattacks by analyzing anomalous spatiotemporal patterns in high-volume, homogeneous network traffic. In contrast, the proposed RESTADM model is designed for the distinct challenges of sensor networks, where data is inherently multi-modal and heterogeneous. In such environments, anomalies extend beyond network-layer cyberattacks to include physical disturbances, device malfunctions, and environmental variations. RESTADM combines Transformer-based temporal modeling with an RBF neural network to enhance sensitivity to localized nonlinear patterns in multi-source, multivariate sensor data. Unlike network traffic analysis that focuses on packet-level or session-level features, our approach processes continuous physical measurements from heterogeneous sensors, requiring specialized handling of cross-domain data heterogeneity and fine-grained abnormal behavior patterns. Therefore, although both studies employ hybrid deep learning architectures, Yuan et al.’s [19] work concentrates on cyberattack classification in network traffic, whereas RESTADM extends the hybrid modeling concept to multi-source sensor anomaly detection, emphasizing the identification of physical and operational anomalies that are characteristic of IoT and sensor network systems.

Although deep learning methods have significantly enhanced anomaly detection performance, sensor networks still encounter numerous challenges in practical applications. Firstly, sensor data typically exhibits high noise levels, high dimensionality, and temporal correlations, which hinder models from accurately identifying anomalous behaviors [20]. Secondly, anomalous data represents an extremely small proportion of data in real-world systems, resulting in severe data imbalance that leads detection models to ‘overlook’ anomalies. Additionally, acquiring high-quality labeled data is challenging, particularly due to the scarcity of anomaly event samples in sensor systems, which further restricts the generalization ability of deep learning models [21].

To address the aforementioned challenges, this paper proposes RESTADM for sensor networks. The model integrates a radial basis function neural network structure within the Transformer framework, employing nonlinear mapping to project complex sensor features into a high-dimensional space, thereby enhancing the model’s sensitivity to subtle anomaly patterns. Concurrently, by combining multi-head attention mechanisms it models feature correlations through similarity scoring and reconstruction error, effectively enhancing the accuracy and robustness of the anomaly detection. Experimental results demonstrate that the RESTADM model achieves superior performance across multiple real-world sensor datasets, significantly reducing false alarm rates and improving detection efficiency, thereby offering an innovative solution for establishing secure and reliable sensor network systems.

## 2. Sensor-Based Data Acquisition and Information Collection

This section details the comprehensive methodology for data acquisition and preparation, which forms the empirical foundation for training and evaluating the proposed RESTADM model. We begin by outlining the overall design of the data acquisition system in Section 2.1. Following this, Section 2.2 describes the specific sensor components employed and the multi-stage information acquisition process. To provide a concrete illustration of the output, Section 2.3 presents a sample of the multi-point data collected. Finally, Section 2.4 explains the crucial steps of data preprocessing and label generation used to construct the final, model-ready dataset.

### 2.1. Design of Data Acquisition System

To construct a high-precision network anomaly detection model, this paper designed and implemented a multi-dimensional sensor information collection system, establishing a comprehensive closed-loop process that encompasses sensor hardware deployment, raw information acquisition, data construction, and annotation. The collected data covers multiple dimensions, such as node operating status, physical environment characteristics, and communication behavior, thereby ensuring that model training is based on a robust data foundation and possesses practical applicability.

This collection system was deployed across multiple intelligent nodes in a heterogeneous environment, characterized by high universality and stability. Each node integrated various hardware sensor modules and leverages edge computing alongside remote communication mechanisms to enable dynamic sensing and real-time uploading of node operating status and environmental characteristics.

The system was deployed for a total of 30 days with a sampling period of 5 min, covering multiple typical working conditions, including normal operation status, communication-intensive periods, node temperature rise stages, as well as abnormal events. Each record was accompanied by a timestamp and node number, thus ensuring data traceability and structural integrity.

### 2.2. Sensor Composition and Information Acquisition Process

In the data collection platform of this study, in order to comprehensively reflect the operating status of the sensor nodes and variations in the external environment, the system integrates diverse sensor modules, encompassing dimensions such as environmental monitoring, behavioral perception, and network monitoring. These sensors possess high stability, compact size, rapid response, and are easily integrated with embedded platforms, facilitating large-scale deployment.

**Figure 1:**
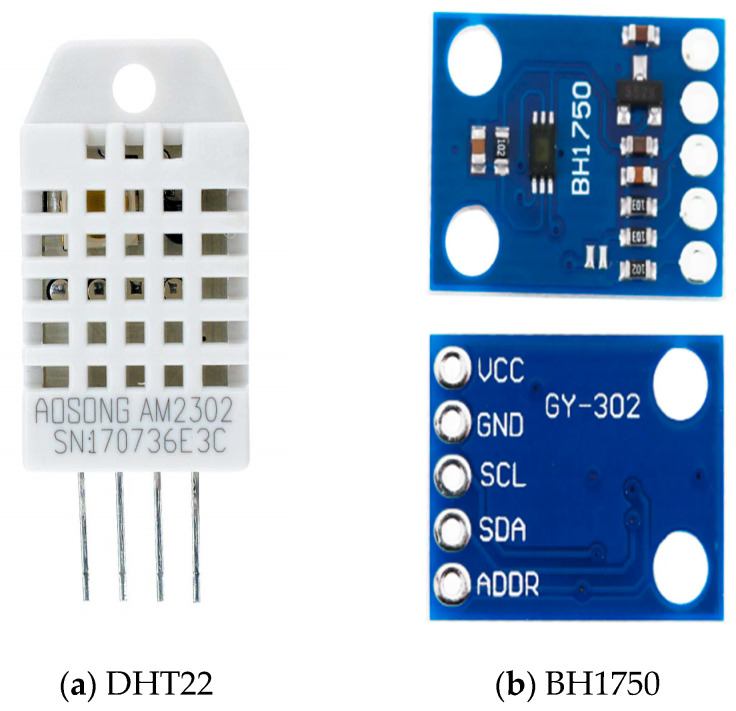

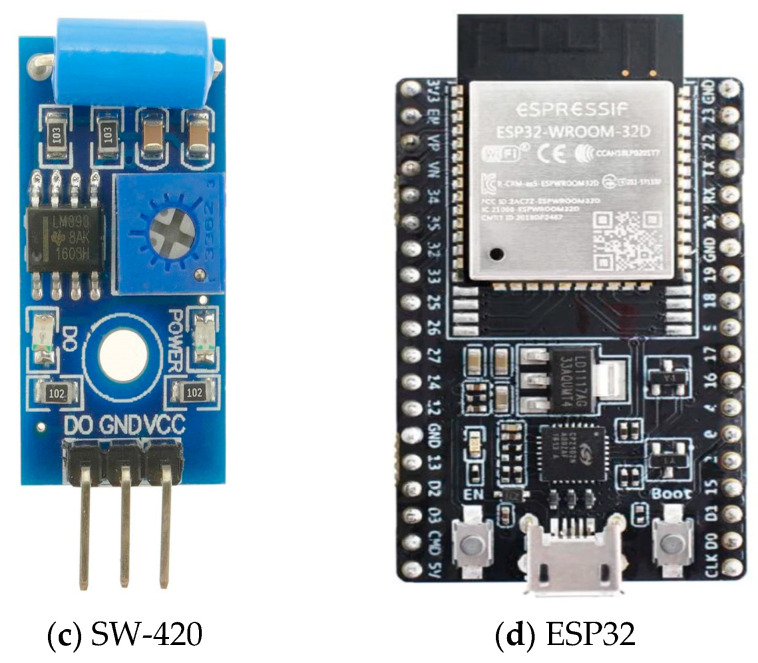
Representative sensor modules employed in the acquisition system: (**a**) DHT22 temperature and humidity sensor, (**b**) BH1750 light sensor, (**c**) SW-420 vibration sensor, (**d**) ESP32 Wi-Fi module.

In the experiment, the DHT22 (AOSONG, Guangzhou, China) temperature and humidity sensor, as shown in Figure 1a, was used to monitor changes in temperature and humidity within the node’s environment, thereby aiding in the identification of abnormal sensor states resulting from physical environmental anomalies. The BH1750 light sensor (Rohm Semiconductor, Kyoto, Japan), as shown in Figure 1b, was employed to detect environmental light intensity, which can indirectly indicate whether the node is experiencing occlusion, malfunction, or abnormal operation. The SW-420 vibration sensor (manufactured by various suppliers, commonly sourced from Shenzhen, China), as shown in Figure 1c, was utilized to detect device vibrations, thereby effectively identifying potential human intervention, accidental collisions, or positional changes. Wireless communication traffic was captured using an ESP32 module (Espressif Systems, Shanghai, China) equipped with Wi-Fi Sniffer functionality, as shown in Figure 1d. This module enables real-time monitoring of inter-node communication traffic, captures underlying data packets, and provides essential data for analyzing abnormal network-layer behaviors.

All aforementioned sensors were connected to the edge computing node via UART or I2C communication protocols, with collected data uploaded to the cloud server through MQTT for centralized storage and unified processing. In total, five sensor nodes were deployed in the experimental setup, each integrating temperature, humidity, illumination, and vibration sensors, along with network traffic monitoring modules.

The information acquisition process was divided into four stages. First, the acquisition of raw signals, during which node status was periodically collected through the sensors. Second, edge processing was performed, utilizing local devices for outlier removal and data normalization. Third, protocol uploading was executed, with data streams transmitted via lightweight protocols (e.g., MQTT). Finally, cloud aggregation occurred, with the server uniformly formatting, labeling, and structuring the data.

### 2.3. Multi-Point Data Collection Sample Display

To provide an intuitive representation of the data features acquired by this system, Table 1 presents sensor data samples from three representative nodes over a selected collection period. The data exhibits both time-series and multi-dimensional characteristics. Anomaly labels are manually annotated by integrating system logs with expert-defined rules, where a label of 0 denotes a normal state and 1 denotes the detection of abnormal behavior or state drift.

As illustrated in Table 1, abnormal events are frequently associated with abrupt changes in several physical indicators, including rapid increases in temperature, abnormal vibration states, and sudden surges in data packet transmission. These changes serve as critical foundations for feature extraction and classification in subsequent anomaly detection models. These anomalies manifest differently across variables. For example, a sudden temperature rise may indicate node overheating, while abnormal humidity levels may lead to corrosion or short-circuiting. Abrupt drops in illumination can reflect occlusion or sensor malfunction, and abnormal vibration states often reveal external disturbances such as collisions or human interference. In terms of network behavior, unexpected surges in packet bytes or irregular protocol distributions may correspond to denial-of-service attacks or unauthorized access attempts. Such diverse manifestations make it difficult for traditional statistical or machine learning methods to capture both local and global dependencies effectively. In contrast, the proposed RESTADM model leverages both the Transformer, to capture temporal dependencies, and the RBF layer, to enhance sensitivity to subtle deviations, making it more efficient and reliable in detecting anomalies across heterogeneous variables.

### 2.4. Data Preprocessing and Label Generation

To improve the training efficiency and detection accuracy of the model, the originally collected data must be systematically pre-processed prior to modeling. Pre-processing steps include the removal or interpolation of missing values and outliers, normalization of physical environment variables, segmentation of network traffic data using a sliding time window approach, and manual annotation of abnormal events by integrating expert knowledge with the existing attack feature library to establish binary classification labels (normal/abnormal). The resulting dataset comprises approximately 200,000 structured records, encompassing diverse abnormal types such as DoS attacks, port scanning, and sensor failures, thereby providing reliable data support for subsequent model training and performance evaluation. Specifically, this labeled dataset, containing both normal and abnormal (e.g., DoS attacks, port scanning) instances, serves as the ground truth for training the RESTADM model to learn discriminative features and for evaluating its detection performance in the experiments described in Section 4.

## 3. RESTADM Model Structure

**Figure 2:**
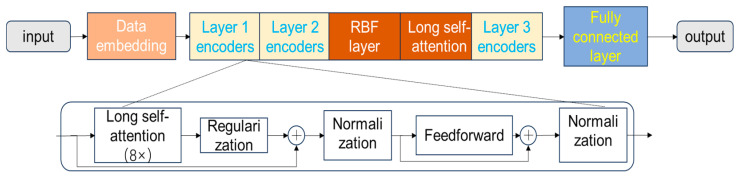
RESTADM Model structure diagram. Arrows indicate the flow of data processing.

Following the data acquisition and preprocessing steps described in Section 2, this section introduces the proposed RESTADM model. The model is designed to learn from the multi-dimensional sensor data to effectively distinguish between normal and abnormal traffic patterns as defined by the generated labels. The model’s design uniquely integrates a RBF neural network within the Transformer framework to enhance anomaly detection sensitivity. We will begin by detailing the role and formulation of the RBF layer in Section 3.1, explaining how it measures the similarity of latent representations to learned patterns. Subsequently, Section 3.2 will elaborate on the Multi-Head Self-Attention mechanism, which is the core component of the Transformer architecture responsible for capturing temporal dependencies and feature correlations within the sensor data. The overall structure, which illustrates how these components are integrated, is depicted in Figure 2.

### 3.1. Radial Basis Function Neural Network

This paper integrates the anomaly detection mechanism into the foundational Transformer architecture through specialized RBF (Radial Basis Function) neurons, as illustrated in Figure 2. The specific placement of the RBF layer after the second encoder layer is a deliberate design choice, motivated by ablation studies (detailed in Section 4.3.3) which indicate that this position yields slightly superior performance compared to other placements. The RBF layer operates on the latent representations from the preceding layer, denoted $H_{i}={\left\{h_{i,t}\right\}}_{t=1}^{T}$, where each $h_{i,t}\in R^{d_{h}}$. It computes similarities between every representation $h_{i,t}$ and a set of M learnable centers $\mathbb{C}={\left\{c_{m}\right\}}_{m=1}^{M}$, with $c_{m}\in R^{d_{h}}$. This yields the RBF outputs ${\mathbb{Z}}_{i}={\left\{z_{i,t}\right\}}_{t=1}^{T}$, where $z_{i,t}\in R^{M}$. These outputs are then passed to the subsequent layers. The similarity between each data point and each center is defined as:

(1) $$z_{i,t}^{m}\left(h_{i,t},c_{m}\right)=\exp\left(-\frac{1}{2}e^{\gamma }{\left\|h_{i,t}-c_{m}\right\|}^{2}\right)$$

In this context, the parameter γ, the parameter that controls the width of the RBF kernel, fundamentally shapes how the RBF layer evaluates data points relative to their respective centers. Intuitively, γ determines the sensitivity of each RBF neuron: a larger γ value leads to a more localized and specialized kernel (a smaller “width”), which is only responsive to inputs that are very close to its center. Conversely, a smaller γ results in a broader, more generalized kernel (a larger “width”) that is influenced by a wider range of inputs. The exponential transformation involving γ also ensures that the scale parameter remains positive, which simplifies the optimization process.

To further enhance the anomaly detection capability, we introduced an RBF layer after the second encoder layer of the Transformer. The rationale was that while the self-attention mechanism excels at capturing global temporal dependencies, it may overlook localized nonlinear variations that are critical for distinguishing subtle anomalies. The RBF layer provides localized feature mapping with strong nonlinear approximation capability, thereby complementing the global context modeling of the Transformer. This design choice enabled RESTADM to achieve a balance between global dependency modeling and local anomaly sensitivity. The proposed RESTADM model was trained by minimizing the mean squared error (MSE) to achieve precise reconstruction. For anomaly detection, a comprehensive anomaly score, referred to as the RESTADM score, was introduced. This score is obtained by combining the normalized RBF similarity score with the reconstruction error. The normalization operation is based on the Min-Max normalization method [22], to ensure comparability, the RBF similarity score is used to measure $x_{i,t}$ which represents the match with the learned center points. The higher the similarity, the more normal the behavior is deemed to be, whereas a lower similarity (or a greater distance from the RBF center) indicates an anomaly. This score is derived by aggregating the RBF outputs across all center points. $z_{i,t}$ by averaging the values obtained. The reconstruction error is calculated as the discrepancy between the actual data $x_{i,t}$ and its reconstructed value ${\overset{\wedge }{x}}_{i,t}$ the squared difference between them. The RESTADM score is calculated using the following formula:

(2) $$RESTADM_{score}\left(x_{i,t}\right)=\varepsilon_{r}\times \varepsilon_{s}$$

Among them $\varepsilon_{r}={\left\|x_{i,t}-{\overset{\wedge }{x}}_{i,t}\right\|}^{2}$ is represented as reconstruction error $\varepsilon_{s}=\left(1-\frac{1}{M}\sum_{m=1}^{M}z_{i,t}^{m}\right)$ It is used to gauge discrepancies. This combined method highlights subtle anomalies with both low reconstruction error and low RBF scores, alongside significant anomalies featuring either high reconstruction error or low RBF scores.

Proper initialization of the RBF parameters, including center parameters and scale parameters, is essential to the methodology proposed in this study. To address this, two initialization strategies—random initialization and K-means initialization—are explored to assess their impact on model performance. For random initialization, the parameters are drawn from a normal distribution with a mean of zero and a standard deviation of one. Despite its simplicity, this approach may lead to slower convergence, an increased risk of convergence to local minima, and an initial inability to effectively represent the data distribution, which may result in model instability. In contrast, K-means initialization leverages the inherent structure of the data to obtain a more representative initialization. In this approach, a base model (excluding the ensemble radial basis function layer) is initially trained to minimize the mean squared error of reconstruction:

(3) $$ΜSE=\frac{1}{N}\sum_{i=1}^{N}{\left\|{Χ}_{i}-{\overset{\wedge }{Χ}}_{i}\right\|}_{F}^{2}$$

where ΜSE denotes the mean squared reconstruction error; ${Χ}_{i}$ represents the ground-truth value of the i-th sample; ${\overset{\wedge }{Χ}}_{i}$ denotes the reconstructed output corresponding to ${Χ}_{i}$; and N is the total number of samples. The operator ‖⋅‖ refers to the Euclidean norm, which measures the squared distance between the original and reconstructed feature vectors.

After the base model achieves satisfactory reconstruction accuracy, latent representations are extracted from specific layers where the RBF layer is intended to be integrated subsequently. These latent representations are then utilized to initialize the center points via the K-means clustering algorithm, while the scale parameters are initialized, γ is translated as ${\tilde{\sigma }}^{2}$ to perform initialization; ${\tilde{\sigma }}^{2}$ is the mean squared distance from each data point to its nearest cluster center. The formula is as follows:

(4) $${\tilde{\sigma }}^{2}=\frac{1}{NT}\sum_{i=1}^{N}\sum_{t=1}^{T}\underset{m}{\min}{\left\|h_{i,t}-c_{m}\right\|}^{2},\forall m\in \left[1,M\right]$$

Among them, $h_{i,t}$ denotes the latent representation vector of the *i*-th (sample/entity) at the *t*-th time step, while $c_{m}$ is obtained via the K-means algorithm as the m-th cluster center. The value of ${\tilde{\sigma }}^{2}$ is used to compute $\gamma =\frac{1}{{\tilde{\sigma }}^{2}}$, and ${\tilde{\sigma }}^{2}$ is initialized based on the average dispersion of data points around their respective centers to ensure that each RBF determines its scope of influence appropriately.

### 3.2. Multi-Head Self-Attention Module

**Figure 3:**
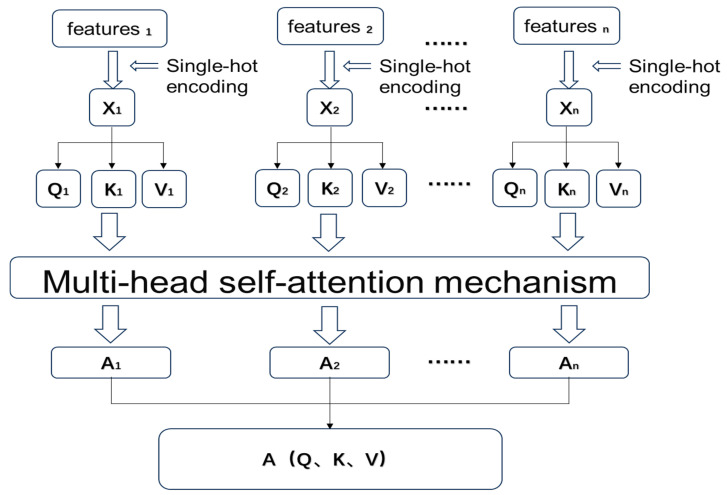
Multi-head attention for traffic anomaly detection. The arrows indicate the flow of data processing and feature transformation.

The inputs to this module are derived from the preprocessed sensor traffic data described in Section 2.3 and Section 2.4, including environmental parameters (temperature, humidity, illumination), vibration states, and network traffic features (e.g., packet bytes, protocol type) with their corresponding anomaly labels. As illustrated in Figure 3 below, the application of the self-attention mechanism in anomaly traffic detection is presented. Given that a feature is a scalar value, while the computation of the self-attention mechanism is based on matrix operations, it is necessary to transform each feature into a vector form. As shown in Figure 3, assuming a traffic flow contains m individual features, each feature is transformed into a feature vector (F) using one-hot encoding, resulting in the feature representations $x_{1},x_{2},\ldots ,x_{m}$. Specifically, categorical attributes such as protocol type and anomaly labels are transformed using one-hot encoding, whereas continuous attributes such as temperature, humidity, and illumination are normalized to ensure comparability across different scales. This unified representation guarantees that both discrete and continuous features are mapped consistently into the feature space before applying the self-attention mechanism. After converting each feature into a one-hot encoded feature vector, the self-attention mechanism is employed to compute the similarity between these F, thereby learning the interrelationships among the features. This is achieved through the parallel computation of attention values (*A*) that quantify the relevance among feature vectors. The resulting attention matrix represents the learned dependencies between the different features. $\left(A_{1},A_{2},\ldots \ldots ,A_{m}\right)$, the final output is an A. $A\left(Q,K,V\right)$, by leveraging this matrix, the model can learn the relationships between features, thereby achieving the objective of detection.

**Figure 4:**
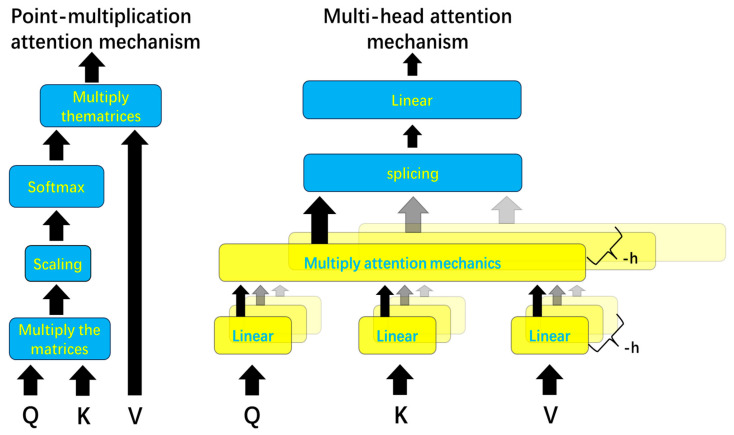
Comparison between dot-product attention mechanism and multi-head attention one. Arrows indicate the flow of data processing; the thick black arrows represent the main data flow, while the thin gray arrows indicate the multiple parallel heads (h). The different colored blocks are primarily used for visual distinction and grouping of the functional layers.

To improve the learning of interrelationships among the features, this study employed the multi-head attention mechanism, which extends the self-attention mechanism. The self-attention mechanism was originally termed the scaled dot-product attention mechanism by the Google research team [23]. Figure 4 illustrates the differences between the scaled dot-product attention mechanism and the multi-head attention mechanism.

The calculation method of multi-head attention builds upon the single-head approach by applying separate linear transformations to the query (Q), key (K), and value (V) matrices. Taking Q as an example, assuming the number of heads is h, splitting *Q* into h part $\left(Q_{1},Q_{2},\ldots ,Q_{h}\right)$, sf *Q* has dimensions M×N, then the dimensions of $Q_{1},Q_{2},\ldots ,Q_{h}$ transform M/h×N (Here, the value of *h* must divide *M* evenly for *Q*, and similarly for *K* and *V*.) *Q*, *K*, and *V* are transformed into multi-head representations via linear projections, with the computation formulated as follows:

(5) $$Q_{h}=Q\times W_{h}^{Q}$$

(6) $$K_{h}=K\times W_{h}^{K}$$

(7) $$V_{h}=V\times W_{h}^{V}$$

Among them $W_{h}^{Q}$, $W_{h}^{K}$ and $W_{h}^{V}$ The parameter matrices represent the linear transformations used in the multi-head attention mechanism. The primary improvement of this mechanism lies in applying multiple distinct linear transformations to the query vectors (*Q*), key vectors (*K*), and value vectors (*V*). This allows the model to compute attention in parallel across different subspaces, thereby enhancing its ability to capture complex relationships and features. Within each head, *Q*, *K*, and *V* are mapped into a new linear space, and the attention mechanism then calculates the correlations between the vectors within this new space. Each head can focus on different types of information; for example, some heads may emphasize local features, while others may concentrate on global patterns.

An important characteristic of the multi-head attention mechanism is that, despite performing multiple linear transformations, the final result—after concatenation and a subsequent linear transformation—can be mapped back to the same dimensionality as the original input. This means that the output dimension of the multi-head attention mechanism is structurally consistent with that of the single-head attention mechanism, and therefore, it does not increase the model’s complexity or introduce additional dimensions. This concatenation process across multiple heads effectively integrates attention results from diverse perspectives, rather than merely adding redundant information. This feature enables the multi-head attention mechanism to enhance the model’s expressive capacity without substantially increasing computational complexity.

In the multi-head attention mechanism, each head independently computes the dot-product between *Q*, *K*, and *V* to calculate its respective attention weights. This process is essentially the same as the dot-product attention mechanism, where the dot product between *Q* and *K* is used to measure the correlation between each position in the input sequence, followed by a weighted summation of *V* to generate the final representation for each position. The key distinction lies in the fact that the multi-head mechanism does not rely solely on a single attention computation; instead, it calculates attention weights across multiple subspaces in parallel through different heads. This allows the model to capture diverse relationships between features across different subspaces, thereby enhancing its ability to model complex patterns and dependencies in the data.

**Figure 5:**
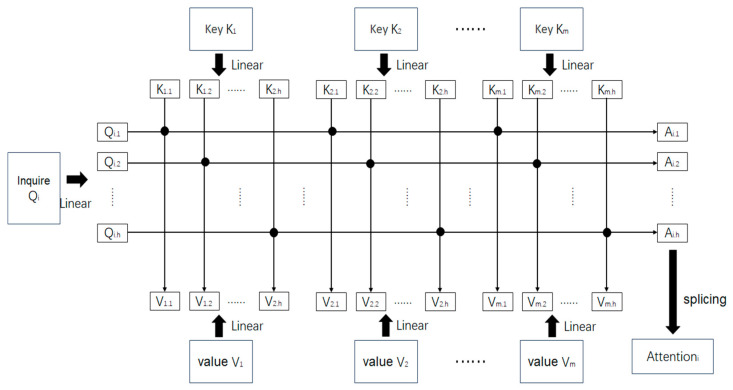
Multi-head attention calculation steps. The figure illustrates the parallel calculation across multiple attention heads (h) for m features. Arrows indicate the flow of data processing. The black dots at the intersection of Query and Key lines represent the dot-product operation used to calculate attention weights.

As shown in Figure 5, the computation method of the multi-head attention mechanism is introduced. Suppose there are m features in a traffic flow, $\left(Q_{1},K_{1},V_{1}\right),\ldots ,\left(Q_{m},K_{m},V_{m}\right)$ corresponding to each feature, respectively, the number of heads is h. Taking the i feature’s query $Q_{i}$ as an example. First, Q, K, V are each transformed through a linear transformation into multiple heads. $Q_{i}$ is transformed into $Q_{i,1},\;Q_{i,2},\;\ldots ,\;Q_{i,h}$; K, V are transformed similarly. Taking the first head of $Q_{i}$ as an example, the query $Q_{i,1}$ is multiplied with the value $V_{1,1},V_{2,1},\ldots ,V_{m,1}$ and then scaled, the weight values are obtained through the softmax, subsequently, a weighted sum is computed with $V\_\left\{1,1\right\},V\_\left\{2,1\right\},\ldots ,V_{m},1$ to obtain the attention value $A_{i,1}$. The multi-head formula is as follows:

(8) $$head_{h}=Attention\left(Q_{h},K_{h},V_{h}\right)$$

(9) $$MultiHead\left(Q,K,V\right)=Concat\left(head_{1},head_{2},\ldots ,head_{h}\right)W^{o}$$

The improvement of the multi-head attention mechanism lies in applying multiple distinct linear transformations to the query Query,Key,Value, the attention values are calculated separately for each linear transformation, and the results are then concatenated. This approach effectively enhances the model’s fitting capacity. During model training, the parameter matrices for the linear transformations are continuously learned and adjusted. The operation of performing multiple linear transformations is analogous to using multiple convolution kernels in CNNs for multi-channel convolution operations, where different kernels are responsible for extracting distinct features. Similarly, the multi-head mechanism serves to analyze feature relationships from different perspectives, extract correlations, and further improve the model’s fitting capacity by increasing the number of model parameters.

## 4. Experiments and Results Analysis

### 4.1. Experimental Environment and Parameter Configuration

In this study, two types of datasets were used for model evaluation. First, the sensor data collected by our acquisition system described in Section 2 were employed to verify the applicability of RESTADM in a real-world sensing environment. These data samples were used in preliminary offline calculations and case studies to ensure that the model could effectively process heterogeneous multi-source sensor signals. Second, to ensure a comprehensive and reproducible evaluation, our model was tested on two widely used public benchmark datasets for time-series anomaly detection: the Server Machine Dataset (SMD) and the Power Supply Machine (PSM) dataset [24,25,26].

The SMD, sourced from a large internet company, is a large-scale benchmark containing monitoring metrics from 28 server machines. It consists of 38 features and was split into 708,420 samples for training and 708,405 for testing, with an overall anomaly ratio of 4.16%.

The PSM dataset, collected from application servers at eBay, is known for its complexity. It includes 25 features and was divided into 87,842 training samples and 87,841 testing samples, with a higher anomaly ratio of 29.8%.

For both datasets, we followed the standard evaluation protocol where the first 50% of the data is used for training and the remaining 50% is reserved as an independent test set. Crucially, this test set was held out and used only once for the final performance evaluation after the model was fully trained. All metrics reported in this paper were calculated on this completely unseen data to ensure a fair and unbiased assessment of the model’s generalization capabilities. These datasets were selected to validate the generalization ability and robustness of the proposed model under different real-world conditions.

Our proposed RESTADM model was trained using the Adam optimizer with an initial learning rate of 1 × 10^−4^ for a maximum of 100 epochs. The RESTADM model was implemented using Python (v3.9) and the PyTorch (v1.13.1) deep learning framework. As detailed in Table 2, our Transformer architecture is composed of three encoder layers, with a model dimension (d_model) of 512 and eight attention heads. The RBF network component utilizes 128 centers and was initialized using the K-means algorithm. A dropout rate of 0.1 was applied within the Transformer layers to mitigate overfitting. The complete hyperparameter configuration is presented in Table 2.

### 4.2. Evaluation Criteria

This paper employs evaluation metrics commonly used in the field of anomalous traffic detection, including accuracy, precision, recall, and F1-score, to validate the model performance [27]. The confusion matrix is presented in Table 3.

The formulas for precision, accuracy, recall, and F1-score in the context of anomaly detection are defined as follows:

(10) $$Precision=\frac{TP}{TP+FP}$$

(11) $$Recall=\frac{TP}{TP+FN}$$

(12) $$Accuracy=\frac{TP+TN}{TP+FN+FP+TN}$$

(13) $$F1-score=\frac{2\cdot recall\cdot precision}{recall+precision}$$

In practical applications, it is common to adopt different evaluation metrics or thresholds depending on the specific requirements of the task. The Receiver Operating Characteristic (ROC) curve is a powerful tool for evaluating a model’s generalization performance, particularly in binary classification problems such as anomaly detection. In addition to standard evaluation metrics, we plotted the ROC curve of the RESTADM model on both the SMD and PSM datasets. The ROC curve illustrates the trade-off between the True Positive Rate (TPR) and False Positive Rate (FPR) at different decision thresholds. The corresponding Area Under the Curve (AUC) values quantify the overall detection capability of the model, with higher AUC values indicating stronger discriminative power.

**Figure 6:**
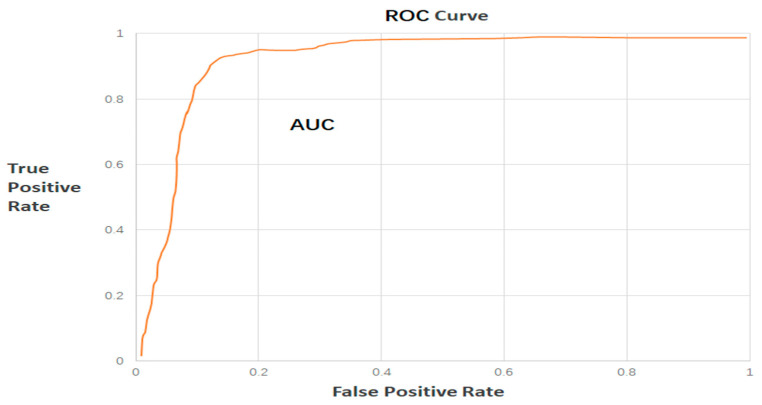
ROC curves and AUC values of the RESTADM model on the SMD and PSM datasets. The curves are derived from experimental results and illustrate the trade-off between detection sensitivity and false alarm rate.

Figure 6 presents the ROC curve and the corresponding AUC. The True Positive Rate (TPR) is placed on the vertical axis, while the False Positive Rate (FPR) is placed on the horizontal axis to construct the ROC curve. The axes are defined in Equations (14) and (15). The AUC represents the area under the ROC curve and is commonly used in practice to effectively evaluate model performance. A model performs better when the AUC is closer to one, whereas an AUC value of 0.5 reduces the ROC curve to a diagonal line, indicating that the model has degenerated into random guessing [28].

(14) $$TPR=\frac{TP}{TP+FN}$$

(15) $$FPR=\frac{FP}{TN+FP}$$

### 4.3. Result Analysis

#### 4.3.1. Baseline Result Analysis

**Figure 7:**
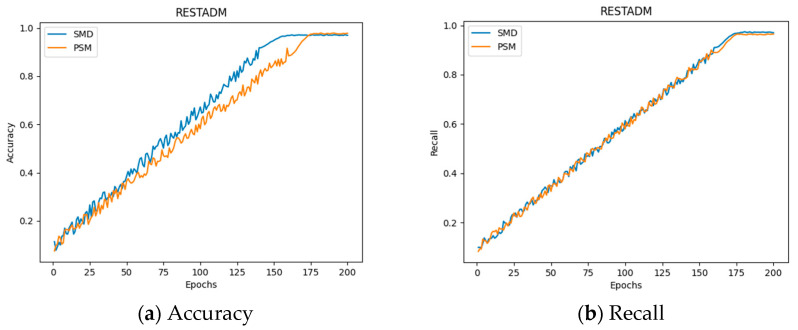

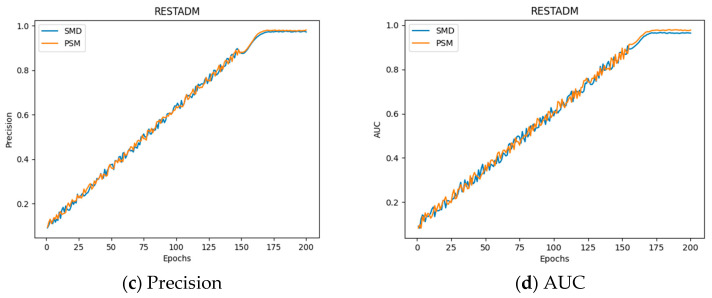
Analysis of RESTADM model results.

Figure 7 presents the RESTADM model’s performance metrics, including accuracy, precision, recall, and AUC (Area Under the ROC Curve).

Figure 7 shows that during training, as the model was continuously optimized, the accuracy, precision, recall, and AUC metrics gradually improved and stabilized around the 175th epoch. Both datasets exhibited fluctuations during the early stages but eventually converged to relatively high values. This indicates that both datasets are effective for learning anomalous features from traffic data. Furthermore, Figure 7a shows that the precision of the SMD is slightly higher than that of the PSM dataset, suggesting that the RESTADM model performs better on the SMD and is more effective at extracting features for detection and classification.

#### 4.3.2. Comparative Experimental Analysis

In the comparative experiment, this study employed several commonly used models for anomalous traffic detection, such as CNN, LSTM, BiLSTM, and others, to compare their detection performance. The detection performance of different models across various datasets is presented in Table 4.

Table 4 shows that the proposed model performs well on both the SMD and PSM datasets. On the SMD, it demonstrates considerable advantages in recall, precision, and F1-score compared to baseline models such as RNN and LSTM, although it performs slightly worse in accuracy and AUC than the Transformer. This may be due to the proposed model forgoing the marginal benefits of stacking complex Transformer layers. Nevertheless, the overall performance of the proposed model remains comparable to that of the Transformer and other models. On the PSM dataset, while the proposed model performs slightly worse than Bi-LSTM and Transformer in recall, it significantly outperforms the baseline models in terms of accuracy, precision, F1-score, and AUC, indicating its strong generalization capability. To ensure the statistical reliability of the reported improvements, each experiment was independently repeated five times, and the results in Table 4 are reported as mean ± standard deviation. We further conducted paired *t*-tests between RESTADM and Transformer for each evaluation metric. The results show that RESTADM consistently outperforms Transformer with statistically significant improvements (*p* < 0.05 or *p* < 0.01), confirming that the observed gains are not due to random variation.

#### 4.3.3. Ablation Experiment Analysis

**Figure 8:**
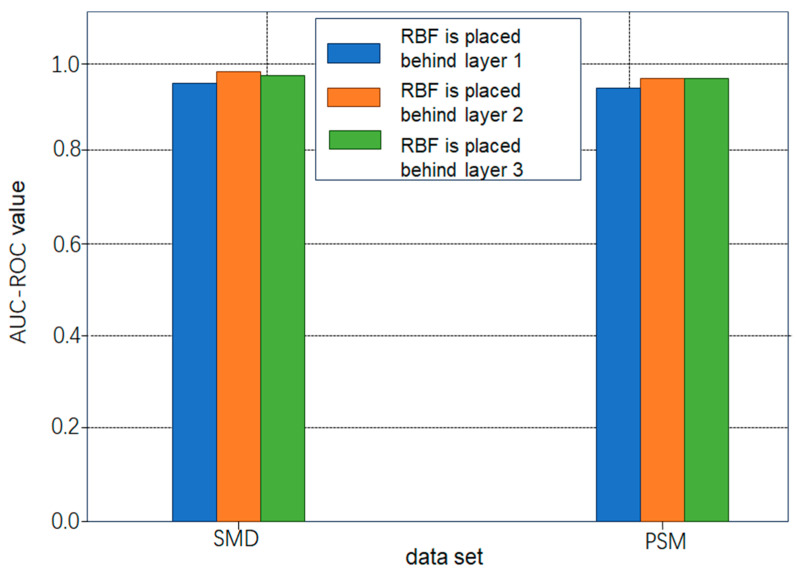
Performance of RESTADM with RBF layer at different positions.

The ablation experiments were conducted using a randomly initialized RBF layer. This study examined the flexibility of placing the RBF layer after each of the three encoder layers in a conventional Transformer. Figure 8 illustrates that, regardless of its position, performance remains stable across datasets. However, placing the RBF layer after the second encoder layer results in slightly better performance. This marginal advantage motivated the placement of the RBF layer after the second layer in the final model architecture (as shown in Figure 2).

In addition to the detection performance, we also examined the complexity of RESTADM relative to baseline models. RESTADM extends the Transformer structure by integrating an RBF neural network layer, which introduces only a small number of additional parameters. The computations of the RBF layer are vector-level operations, leading to limited extra overhead during training. When trained under the same experimental environment as described in Section 4.2, RESTADM required slightly longer training time compared with the Transformer baseline, but the increase remained modest and acceptable. Considering the substantial improvement in anomaly detection performance, the additional computational cost introduced by RESTADM can be regarded as a reasonable trade-off. Compared with the pure Transformer baseline, RESTADM achieved consistent improvements across precision, recall, and F1-score on both SMD and PSM datasets. More importantly, the integration of the RBF layer contributes to robustness against heterogeneous anomalies, including both cyberattacks and physical sensor malfunctions. This indicates that the theoretical advantage of combining global attention with localized RBF mapping translates into practical performance gains.

## 5. Conclusions

This paper proposes a novel sensor network anomaly traffic detection model, RESTADM, which integrates Transformer and RBF techniques. The model leverages the Transformer architecture to effectively capture dependencies in sensor time-series data, while the RBF neural network enhances the perception of subtle anomaly patterns. Furthermore, a multi-head self-attention mechanism is introduced to model complex interactions among sensor features. Experimental results demonstrate that RESTADM achieves superior detection performance across multiple publicly available sensor datasets, particularly outperforming current state-of-the-art anomaly detection methods in terms of recall and F1-score, while also exhibiting strong accuracy and robustness.

While the RESTADM framework draws conceptual inspiration from hybrid architecture designs, it is essential to clarify that the model is not intended for direct application to network cyberattack detection. RESTADM is specifically designed and optimized for sensor network environments where anomalies primarily manifest as deviations in physical measurements and operational parameters, rather than malicious network traffic patterns. The model’s input features consist of multi-modal sensor readings (temperature, humidity, vibration, light intensity), not the network-level attributes (packet features, flow statistics, protocol signatures) typically required for cyberattack identification. This specialization serves a distinct application domain focused on the physical and operational integrity of sensor systems.

Despite these advantages, the RESTADM model presents certain limitations. Its relatively complex structure demands substantial computational resources during training, and the initialization and parameter selection of the RBF layer significantly influence detection outcomes. These factors increase the difficulty of deployment and the cost of optimization. Future research could therefore focus on designing lightweight architectures, improving training efficiency, and incorporating automated hyperparameter tuning techniques. Additionally, exploring RESTADM’s generalization ability and evaluating its practical performance across diverse sensor network scenarios could further enhance its applicability and reliability. Although RESTADM demonstrates competitive performance on benchmark datasets, several practical limitations should be acknowledged. First, the model relies on high-quality labeled data for training, and the annotation of physical anomalies and cyberattack events may be costly or infeasible in real-world environments. Second, while computational complexity has been addressed, the model’s applicability to resource-constrained edge devices remains to be further validated, particularly regarding inference latency and energy consumption. Third, the diversity of anomalies in practical sensor networks is often greater than that represented in the benchmark datasets, which may limit the generalization ability of the model. These limitations indicate that future research should investigate lightweight model structures, online or incremental learning strategies, and cross-domain adaptation mechanisms to improve real-world deployment feasibility.

## Figures and Tables

**Table 1:** Multi-node sensor data samples (partially shown).

Node Number	Temperature (°C)	Humidity (%)	Illumination (Lux)	Vibration State	Byte	Protocol Type	Abnormal Label
Node-A1	38.1	57.2	370	0	128	TCP	0
Node-A2	39.2	59.1	360	0	1024	UDP	0
Node-A3	40.5	61.0	320	1	980	TCP	1
Node-A1	38.5	57.5	375	0	110	TCP	0
Node-A2	41.0	60.2	355	1	1500	UDP	1
Node-A3	39.9	58.3	365	0	10,400	TCP	0

**Table 2:** Experimental environment and parameter settings.

Hyperparameter	Value
Optimizer	Adam
Learning Rate	1 × 10^−4^
Epochs	100
Dropout Rate	0.1
Number of Encoder Layers	3
Model Dimension (d_model)	512
Number of Attention Heads	8
Feed-Forward Dimension (d_ff)	2048
Number of RBF Centers (M)	128
RBF Initialization Method	K-means

**Table 3:** Confusion matrix for anomaly evaluation results.

True Situation	Predicted Positive	Predicted Negative
Positive Instance	TP	FN
Negative Instance	FP	TN

**Table 4:** Comparative experimental results of different models %.

Model	Precision	Recall	Accuracy	F1-Score	AUC
SMD					
CNN	92.58 ± 0.43%	88.95 ± 0.52%	91.94 ± 0.40%	90.42 ± 0.44%	91.36 ± 0.41%
RNN	85.46 ± 0.47%	89.17 ± 0.49%	77.50 ± 0.55%	82.92 ± 0.51%	83.26 ± 0.53%
LSTM	83.59 ± 0.45%	83.28 ± 0.43%	88.66 ± 0.46%	85.88 ± 0.47%	87.66 ± 0.44%
Bi-LSTM	96.60 ± 0.29%	96.80 ± 0.30%	96.99 ± 0.27%	96.89 ± 0.31%	96.58 ± 0.28%
Transformer	97.69 ± 0.25%	96.35 ± 0.27%	96.57 ± 0.23%	97.39 ± 0.24%	96.87 ± 0.26%
**RESTADM**	97.36 ± 0.22% *	97.22 ± 0.24% *	97.03 ± 0.21% *	98.56 ± 0.23% **	96.55 ± 0.22%
PSM					
CNN	86.59 ± 0.41%	90.35 ± 0.46%	86.85 ± 0.39%	88.57 ± 0.44%	87.36 ± 0.42%
RNN	84.69 ± 0.48%	77.49 ± 0.50%	89.17 ± 0.47%	82.92 ± 0.49%	80.78 ± 0.46%
LSTM	83.54 ± 0.46%	85.90 ± 0.45%	79.51 ± 0.44%	82.58 ± 0.47%	81.67 ± 0.43%
Bi-LSTM	95.48 ± 0.32%	96.99 ± 0.34%	95.24 ± 0.30%	96.11 ± 0.33%	96.14 ± 0.31%
Transformer	97.66 ± 0.26%	96.86 ± 0.28%	95.58 ± 0.25%	96.41 ± 0.27%	96.42 ± 0.26%
**RESTADM**	97.88 ± 0.23% *	96.36 ± 0.24%	97.69 ± 0.22% **	97.70 ± 0.23% **	97.78 ± 0.22% *

Note: Results are reported as mean ± standard deviation over five independent runs. A paired *t*-test was conducted between RESTADM and Transformer. Asterisks (*) and (**) denote statistical significance at *p* < 0.05 and *p* < 0.01, respectively.

## Data Availability

The data that support the findings of this study are available from the corresponding author upon reasonable request.
